# Double trouble: a comprehensive study into unrelated genetic comorbidities in adult patients with Facioscapulohumeral Muscular Dystrophy Type I

**DOI:** 10.1038/s41431-024-01770-0

**Published:** 2025-01-07

**Authors:** Angela Puma, Giulia Tammam, Andra Ezaru, Abderhmane Slioui, Eleonora Torchia, Giorgio Tasca, Luisa Villa, Michele Cavalli, Leonardo Salviati, Patrick J. van der Vliet, Richard JLF Lemmers, Jonathan Pini, Silvère M. van der Maarel, Sabrina Sacconi

**Affiliations:** 1 Peripheral Nervous System & Muscle Department, Pasteur 2 Hospital, Nice University Hospital, Nice, France; 2 Department of Brain and Behavioral Sciences, University of Pavia, Pavia, Italy; 3 IRCCS Mondino Foundation, Pavia, Italy; 4 Università Cattolica del Sacro Cuore, Rome, Italy; 5 John Walton Muscular Dystrophy Research Centre, Newcastle University and Newcastle Hospitals NHS Foundation Trust, Newcastle upon Tyne, UK; 6 Department of Women’s and Children’s Health, Clinical Genetics Unit, University of Padova, Padova, Italy; 7 Department of Human Genetics, Leiden University Medical Center, Leiden, The Netherlands; 8 Institute for Research on Cancer and Aging of Nice, CNRS, INSERM, Côte d’Azur University, Nice, France

**Keywords:** Genetics research, Neuromuscular disease

## Abstract

Facioscapulohumeral dystrophy type 1 (FSHD1) displays prominent intra- and interfamilial variability, which complicates the phenotype-genotype correlation. In this retrospective study, we investigated FSHD1 patients classified as category D according to the Comprehensive Clinical Evaluation Form (CCEF), a category defined by FSHD patients showing uncommon clinical features, to identify genetic causes explaining these uncommon phenotypes. Demographics, clinical data and clinical scales of FSHD1 patients were retrospectively evaluated. Patients were divided into four CCEF categories, and comparisons between groups were performed. In category D, when uncommon features suggested the presence of an unrelated genetic disease, a more extensive collection of data was performed. 157 FSHD1 patients were included in the study (82 males, 75 females) with mean age of 52.1 ± 13.5 years at the time of the study. D4Z4 repeat sizes ranged between 2 and 10 RU. According to the CCEF, 114 patients were classified into category A, 8 into category B and C each, and 27 into category D. In category D, 9 patients presented uncommon features related to commonly acquired comorbidities, whereas in the remaining 18 patients, all but two with upper-sized FSHD1 D4Z4 repeats (7–10 RU), we suspected an unrelated genetic neurological disease based on clinical phenotype. In 14/18 patients, we identified FSHD-unrelated genetic causes, most often unrelated repeat expansion disorders. This emphasizes the need of careful clinical and genetic work-up to avoid confusion between FSHD-intrinsic clinical variability and clinical features unrelated to the disease.

## Introduction

Facioscapulohumeral dystrophy (FSHD: MIM 158900) is one of the most common forms of muscular dystrophy [1], primarily caused by pathogenic contractions of the D4Z4 repeat array on chromosome 4qA to 1–10 repeat units (RU) (FSHD1; MIM 158900). Normally, this repeat array ranges between 8 and 100 RU [2, 3]. Rarely, FSHD can also result from mutations in genes encoding D4Z4-binding chromatin modifiers, such as SMCHD1, DNMT3B, or LRIF1, in combination with at least one 4qA D4Z4 allele of typically 8–20 RU (FSHD2; MIM 158901) [4–6].

In FSHD1, shorter D4Z4 repeats (1–3 RU) are linked to early onset and rapid muscle wasting [7–9], while larger FSHD alleles (7–10 RU) often show high non-penetrance [10, 11]. FSHD2 clinical variability is influenced by D4Z4 repeat size and the specific SMCHD1 mutation, depending on residual SMCHD1 functionality [12]. Both conditions lead to partial D4Z4 chromatin opening and DUX4 gene expression in skeletal muscle, causing similar muscle wasting and atrophy patterns [4, 13, 14]. FSHD typically involves asymmetric muscle weakness starting in facial muscles (*orbicularis oculis* and *oris*) and progressing to shoulder girdle, upper arms, abdomen, and distal lower limbs, with severe cases affecting pelvic and distal upper limb muscles [15]. Respiratory involvement is more common in FSHD2 than FSHD1 [16], and cardiac involvement is rarely symptomatic [17, 18]. Non-muscular features like hearing loss and retinal vasculopathy may occur but are rarely clinically relevant, and cognitive impairment and epilepsy are seen in severe early onset cases [19–21].

Both FSHD1 and FSHD2 exhibit significant intra- and interfamilial variability in onset and progression [13]. These forms overlap in the D4Z4 repeat array size range of 9–10 RU, where a combination of D4Z4 repeat contraction and an FSHD2 gene mutation can lead to a more severe phenotype [5, 22]. Clinical variability in FSHD1 is often influenced by kinship, as the hereditary nature of the disease leads to familial patterns of disease presentation. While family members typically share the same genetic mutation, significant differences in disease severity, age at onset, and progression can be observed within the same family. This variability may be attributed to several factors, including genetic mosaicism, where only a subset of cells carries the D4Z4 repeat contraction [23–26]. In addition, epigenetic factors and other unknown genetic or environmental modifiers may play a role in shaping the phenotypic variability of disease presentation within families. Indeed, clinical variability in FSHD1 has also been suggested to be influenced by factors such as age, sex, hormones, and telomere shortening. [23, 27–30] But, clinical variability in FSHD still remains largely unexplained.

Although the large majority of FSHD patients display a characteristic pattern of muscle involvement, uncommon clinical or paraclinical features may be present in a subgroup of patients. Some may be explained by the coexistence of common comorbidities, but in some cases, they have led to the diagnosis of coexisting neuromuscular genetic diseases, so-called “double trouble” cases [31–38]. These rare findings suggest the possibility that clinical variability in FSHD1 may be related, among other factors, to coexisting unrelated genetic conditions, especially in the presence of uncommon clinical or paraclinical features.

The FSHD Italian national study group has developed a FSHD Comprehensive Clinical Evaluation Form (CCEF) to systematically collect data of patients with FSHD and to classify them according to clinical phenotype into four categories, from A to D, based on the presence of typical and atypical features [39].

To explore whether genetic factors unrelated to FSHD might explain the appearance of uncommon features in FSHD1 patients, we introduced a systematic use of the CCEF to classify FSHD1 patients in daily clinical practice at our center. We carefully studied patients classified in category D, those with uncommon features, as well as their family members, to identify genetic causes responsible for uncommon clinical and paraclinical features. Here we present the results of this retrospective study confirming the hypothesis that FSHD unrelated genetic causes may contribute to the appearance of uncommon features in FSHD1 patients. We describe this subgroup of FSHD patients with uncommon features in comparison to patients with a classical clinical phenotype.

## Materials and methods

This retrospective study was conducted at Nice University Hospital’s Neuromuscular Diseases Reference Center, specializing in diagnosing, following up, and treating adult FSHD.

### Patients

We included patients aged 18 and older with a confirmed FSHD1 diagnosis (1–10 D4Z4 repeats on chromosome 4qA) who were referred to our center between 2017 and 2022, and for whom complete clinical and genetic data (history, family history, age at onset, 4qA D4Z4 repeat size, disease duration, CCEF) were available.

### Genetic analysis

D4Z4 repeat sizing and haplotype analysis for FSHD patients was performed by Southern blot as previously described [40]. Whole Exome Sequencing was performed as reported in [41].

The entire mitochondrial genome was amplified from DNA extracted from peripheral blood leukocytes and skeletal muscle as a single fragment; primers and PCR conditions are available upon request. The amplicon was sequenced on an Illumina MiniSeq sequencer using the Nextera Library Preparation Kit, as reported in [42].

Huntington Disease, Spinocerebellar ataxias type 1, 2, 3, 6, 7, and 8, and Myotonic Dystrophy type 1 were analyzed using the Adellgene Huntington Disease (catalog AD-HD-16), Adellgene SCAs (catalog AD-SCA-16), and Adellgene Myotonic Dystrophy Screening (catalog AD-MD-16) and Confirmatory kits (Catalog AD-MD-C-16, BDR, Zaragoza, Spain), whereas Myotonic Dystrophy type 2 was analysed using Myotonic Dystrophy type 2 SB Kit (code DM.03FL, Clonit, Abbiategrasso, Italy).

### Comprehensive Clinical Evaluation Form (CCEF)

The CCEF (available at http://www.fshd.it/documenti/) has four sections [43]: the evaluation form (clinical history, disability, muscle involvement), the FSHD evaluation scale (score range 0–15), the clinical diagnostic form (summarizes clinical features), and phenotypic classification. Category A includes patients with typical facial and scapular girdle muscle weakness (subcategories A1-A3 by severity); Category B includes patients with weakness limited to scapular girdle or facial muscles (subcategories B1 and B2); Category C includes pauci-symptomatic or asymptomatic patients (subcategories C1 and C2); Category D includes FSHD patients with additional atypical clinical features (subcategories D1 and D2). For patients evaluated multiple times, the most recent CCEF was used.

### Analyses and statistics

We analyzed demographic, clinical, and genetic data for each category, using descriptive statistics. Normality was tested with the Kolmogorov–Smirnov test, and comparisons were made using the Student *t* test or Mann–Whitney test based on normality results. Statistical analyses were conducted using GraphPad Prism7.

For category D patients, who presented with atypical features suggesting a second unrelated disease, we reviewed the medical records for investigations that were performed to evaluate these unrelated diseases, including clinical features, familial history, blood tests, cardiac and respiratory evaluations, muscle biopsies, electromyography, muscle imaging, and genetic testing. Patients who were found to have a second genetic disease were classified as D1w, while those in which a second genetic disease was not found were classified as D1wo. All the analyzed data are included in the Supplementary Data 1.

## Results

### Clinical and demographic data of the study cohort

We included in this study 157 FSHD1 patients (82 male, 75 female). The mean age of the population at the time of the study was 52.1 ± 13.5 years, ranging from 18 to 78 years, with a mean age at symptom onset of 33 ± 14.8 years. In our study cohort, D4Z4 repeats on the disease allele ranged from 2 to 10 RU, with a mean size of 6.5 ± 1.8 (Table 1).

**Table 1:** Clinical, genetics and demographic data of patients included in the study, divided by CCEF categories.

Category / Subcategories	Total N (M/F)	D4Z4 RU	Age at onset (mean ± SD)	Age at the examination (mean ± SD)	Disease duration (mean ± SD)	FSHD score (mean ± SD)	1–3 RU (N)	4–6 RU (N)	7–10 RU (N)
**A**	**114 (59/55)**	**6.1** ± **1.7**	**31.6** ± **13.7**	**51.6** ± **12.6**	**19.9** ± **11**	**7.7** ± **2.6**	**9**	**55**	**50**
A1	24 (12/12)	5 ± 1.5	24.4 ± 8.2	48.8 ± 12.3	24.5 ± 10.4	10 ± 2	4	15	5^a^
A2	63 (33/30)	6 ± 1.7	33 ± 14.4	52.9 ± 13	19.6 ± 11.5	7.5 ± 2.4	5	29	29
A3	27 (14/13)	7 ± 1.4	34.9 ± 13.8	50.9 ± 11.9	16.4 ± 9.1	6 ± 1.9	-	11	16
**B**	**8 (4/4)**	**7.9** ± **1.1**	**42.5** ± **21.2**	**57.3** ± **17.3**	**23.6** ± **17.1**	**2.1** ± **1**	-	-	**8**
B1	5 (3/2)	7.8 ± 0.8	27.6 ± 5.6	51.2 ± 19.5	23.6 ± 17.1	2.6 ± 0.9	-	-	5
B2	3 (1/2)	8 ± 1.7	67.3 ± 5.9	67.3 ± 5.9	NA	1.3 ± 0.6	-	-	3
**C**	**8 (2/6)**	**8.6** ± **1.6**	**54.3** ± **9.9**	**53.4** ± **11.8**	**4** ± **2.4**	**0**	-	**1**^**b**^	**7**
C1	4 (1/3)	7.8 ± 1.7	54.3 ± 9.9	53.4 ± 11.8	4 ± 2.4	0	-	1^b^	3
C2	4 (1/3)	9.5 ± 1	-	47 ± 12.2	-	0	-	-	4
**D**	**27 (17/10)**	**7.6** ± **1.5**	**32.9** ± **15**	**52.3** ± **16.3**	**19.3** ± **10.4**	**7.9** ± **2.1**	-	**6**	**21**
D1w^c^ D1w suspect^d^	14 (10/4) 4 (2/2)	7.9 ± 1.3 7.8 ± 1.7	28.6 ± 16.2 35.8 ± 15.1	46.6 ± 15.3 46.3 ± 16.1	18.1 ± 9.4 10 ± 8.4	8 ± 2.4 7.3 ± 2.1	- -	2^b^ 1^b^	12 3
D1wo^e^	9 (5/4)	6.9 ± 1.6	38.3 ± 12.4	63.8 ± 12.8	25.4 ± 9.6	8 ± 1.9	-	3	6
**Total**	**157 (82/75)**	**6.5** ± **1.8**	**33** ± **14.8**	**52.1** ± **13.5**	**19.5** ± **11.22**	**7** ± **3.1**	**9**	**62**	**86**

^a^all patients with 7 RU.

^b^all patients with 6 RU.

^c^D1w = D1 patients with uncommon features caused by another confirmed genetic disease.

^d^D1w suspect = D1 patients with uncommon features suspect for other genetic disease but not confirmed.

^e^D1wo = D1 patients with uncommon features caused by acquired conditions.

### Characteristics of CCEF-classified FSHD1 patients

**Fig. 1:**
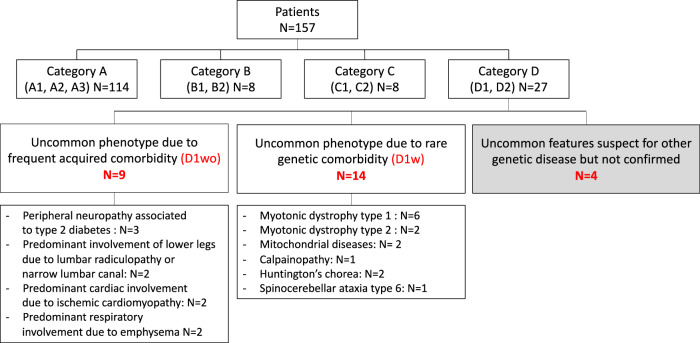
Breakdown of patients with double trouble.

Figure 1 shows a breakdown of the classification for all FSHD patients included. Most patients displayed the classic FSHD phenotype and were classified as category A (N = 114), followed by patients displaying uncommon features classified as category D (N = 27). A minority of patients presented muscle weakness limited to scapular girdle or facial muscles, classified as category B, or were pauci-symptomatic or asymptomatic, being classified as category C (N = 8 each). Of the 114 patients classified in category A, 24 were classified in subcategory A1, 63 in subcategory A2, and 27 in subcategory A3. In categories B and C, patients were equally distributed over both subcategories (5 in B1 and 3 in B2; 4 each in C1 and C2), while in category D, all patients belonged to subcategory D1.

Male and female participants were equally distributed over the different categories, except for category C (C1, C2), in which females prevailed (6/8 females) and category D1 in which male patients were more represented among patients with additional genetic defect (D1w) (10/14 males) (Table 1).

**Fig. 2:**
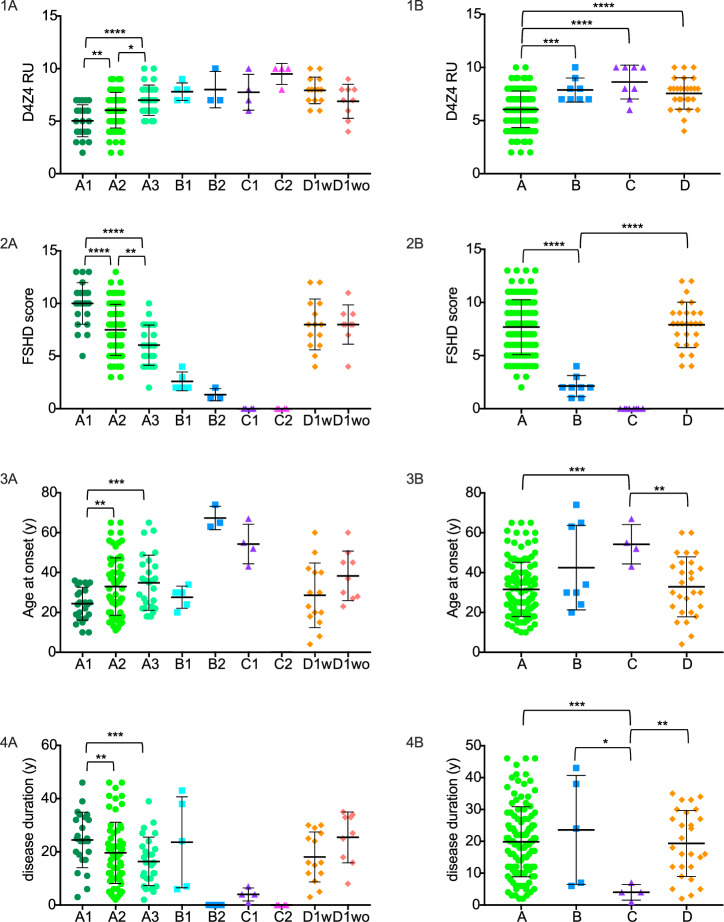
Distribution of D4Z4 repeat units, FSHD score, age at onset, and disease duration in patients divided according to CCEF categories and subcategories. In black, mean and SD values are represented. **1A** Significant differences in D4Z4 size among all A subcategories (****p < 0.0001, **p = 0.01, *p = 0.03); **1B** Significant differences in D4Z4 size between A and B (****p < 0.0003), C (****p < 0.0003) and D categories (***p = 0.002); **2A** Significant differences in FSHD score among all A subcategories (****p < 0.0001, **p = 0.01); **2B** Significant differences in FSHD score between A, B and D categories (****p < 0.0001); **3A** Significant differences for age at onset between A1 vs A2 (***p = 0.003) and vs A3 subcategories (**p = 0.01); **3B** Significant differences for age at onset between C and A (***p = 0.003) and C and D categories (**p = 0.008); **4A** Significant differences for disease duration between A1 and A2 (***p = 0.004), and A1 and A3 subcategories (**p = 0.03); **4B** Significant differences for disease duration between C (***p = 0.0005) and A (*p = 0.03), B and D categories (**p = 0.003).

Regarding category A, patients classified as A1, characterized by severe facial involvement, showed shorter D4Z4 repeat sizes (*p* = 0.01 vs A2 and *p* < 0.0001 vs A3), higher FSHD scores (*p* < 0.0001 vs A2 and vs A3), longer disease duration (*p* = 0.03 vs A2 and *p* = 0.004 vs A3), and earlier age at symptom onset (*p* = 0.01 vs A2 and *p* = 0.002 vs A3) compared to A2 and A3. On the other hand, A3 patients, characterized by less severe facial involvement, had longer D4Z4 repeat sizes (*p* = 0.03) and lower FSHD scores compared to A2 (*p* = 0.01). We found no statistical difference regarding disease duration and age at symptoms onset when comparing A3 with A2 (*p* = 0.3 and *p* = 0.5, respectively) (Fig. 2).

Patients classified as B1, B2, C1 and C2 represented the minority in our cohort, and due to the small number of patients, we did not statistically compare the subcategories of patients within the B and C cohorts, but we considered exclusively the main categories for the analyses. However, we observed that B1 and B2 patients showed similar D4Z4 repeat sizes and FSHD score, with B2 patients presenting lower values (FSHD score ~1), whereas B1 showed an earlier age at onset. All category B patients presented had repeat sizes of 7–10 RU (Table 1, Fig. 2). Regarding category C, the asymptomatic C2 patients presented a longer mean D4Z4 RU. All but one patient belonging to C1 presented with D4Z4 repeat sizes of 7–10 RU (Table 1, Fig. 2).

Category D represented the 17% of our cohort (27/157 patients). All patients belonged to subcategory D1. In 9 patients, we identified a clear relation with an acquired disease responsible for the observed uncommon features (patients without additional genetic defect or D1wo), while in the remaining 18 patients we suspected an unrelated genetic disorder, which we confirmed in 14 patients (patients with additional genetic defect or D1w). We could not find any difference comparing D1w and D1wo patients for D4Z4 repeat units, FSHD score, disease duration and age at onset (Table 1, Fig. 2). For 4 patients we could not achieve a definite genetic diagnosis: two of them had ocular ptosis as uncommon feature, one had prominent dilated cardiomyopathy, and another patient had *pes cavus*. In this patient, the *pes cavus* was not due to a neuropathic involvement. We excluded these patients from further analyses and comparisons between groups.

### Comparisons of CCEF categories (age, disease duration, severity, D4Z4 repeats)

When comparing the different main categories, patients in categories A and D had similar FSHD score (*p* = 0.64), whereas patients in category B had significant lower FSHD score compared to A and D (*p* < 0.0001). D4Z4 size was significantly shorter in category A patients compared to all others (*p* = 0.002 vs B, *p* = 0.0002 vs C, *p* < 0.0001 vs D), while no differences were found when comparing B, C and D (*p* > 0.1). Regarding disease duration and age at symptoms onset, no differences were found when comparing A, B and D categories, but when comparing with category C (subcategory C1), we found a lower age at onset for category A and D (*p* = 0.003 and *p* = 0.008, respectively). Also, disease duration was longer in category A and D patients compared to category C (*p* = 0.0005 and *p* = 0.003, respectively) (Table 1 and Fig. 2).

In each category, patients were also binned into three groups according to the size of the D4Z4 repeats: those with range of 1–3, 4–6 and 7–10 RU. According to this subdivision, the group with 1–3 RU was composed of only 9 patients, all of them belonging to category A, in particular subcategories A1 and A2. The group with 4–6 RU was mainly composed of category A patients, but a minority of them belonged to categories C and D (55 patients from category A, 1 from C and 6 from D1), observing that both the single patient from category C and all D1w patients presented 6 RU. The last group, characterized by 7–10 RU, appeared more heterogeneous, and it was composed of patients belonging to all four categories (50 from category A, 8 from B, 7 from C, 21 from D). Notably, the large majority of D are represented in this range including all but two D1w patients (Table 1).

### Clinical and genetic observations in CCEF category D

We classified 27 patients in category D, specifically subcategory D1, because they also presented with clinical features uncommonly seen in patients with FSHD (Fig. 1). None of the patients belonged to category D2. For 9 of these FSHD patients their uncommon features were clearly related to comorbidities of non-genetic origin (D1wo).

Indeed, three patients had peripheral neuropathy associated to type 2 diabetes; two patients had a predominant weakness in lower legs explained by the coexistence of severe lumbar radiculopathy and narrow lumbar canal; two patients had predominant cardiac involvement secondary to ischemic cardiomyopathy; and finally, two patients had severe respiratory insufficiency associated with emphysema due to high tobacco consumption.

Interestingly, we noted that D1wo patients had a similar mean age at onset, disease duration, size of D4Z4 repeat arrays, and FSHD score compared to category A patients (Table 1). In this group, most patients presented a size of D4Z4 ranging from 7 to 10, while in 3 patients we found 4–6 repeats (Table 1).

Since we could not find any acquired disease explaining the uncommon features in the remaining 18 FSHD patients belonging to category D1, we submitted them to an extensive clinical and diagnostic assessment searching for a possible genetic disease. The assessment varied from patient to patient. While these features were often present in the index case, in some families, clinical and paraclinical examination, symptoms or a history of atypical neurological or muscular presentation in family members prompted genetic testing and helped in identifying the underlying condition (Supplementary material; family 1–6). In other cases, muscle biopsy was of critical value (Supplementary material; family 7, 8).

**Fig. 3:**
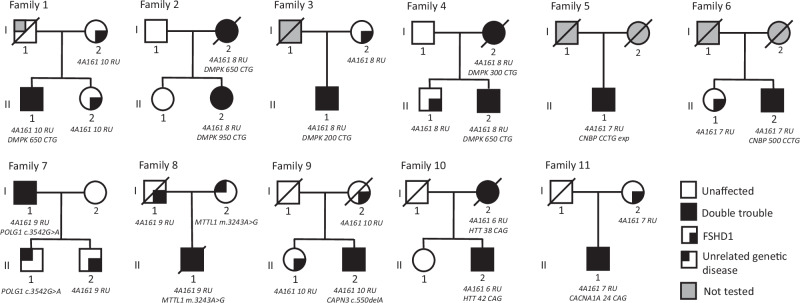
Pedigrees of the eleven families.

In 14 of these FSHD patients, originating from 11 different families, we achieved a genetic diagnosis explaining the uncommon feature (D1w). Among these, we found 6 patients carrying pathogenic expansion in *DMPK* gene associated with myotonic dystrophy type 1 (DM1; MIM 160900). We also identified 2 cases of myotonic dystrophy type 2 (DM2; MIM 602668), 2 patients with mitochondrial disorders, 2 with Huntington’s disease (HD; MIM 143100), one case of FSHD associated with limb girdle muscular dystrophy R1 (LGMD R1/2 A; MIM 253600) and one with spinocerebellar ataxia type 6 (SCA6; MIM 183086) (Fig. 3). The clinical details of these patients and their relatives, when available, are in the supplementary material (Supplementary material).

**Fig. 4:**
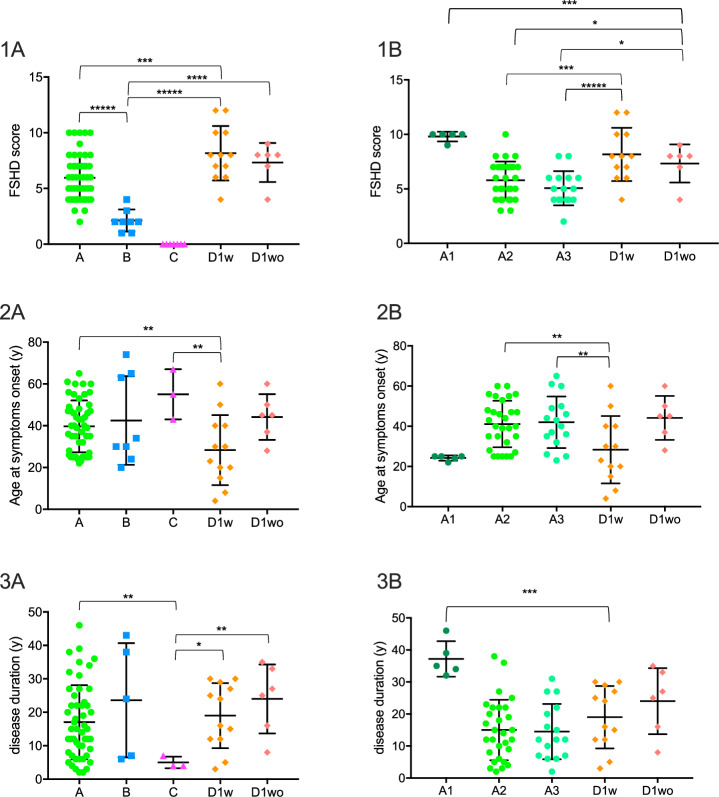
Distribution of FSHD score, age at onset, and disease duration in A, B, C, D1w and D1wo categories and A1, A2, and A3 subcategories with patients carrying 7-10 RU. In black, mean and SD values are reported.**1A** Significant difference in FSHD score between A and B (*****p < 0.0001), A and D1w (***p = 0.005), B and D1w (****p = 0.001), and, B and D1wo (****p = 0.001); **1B** Significant difference in FSHD score between D1w and A2 (***p = 0.003), and A3 subcategories (*****p < 0.0001) and for D1wo compared to A1 (***p = 0.006), A2 (*p = 0.04) and A3, (*p = 0.04); **2A** Significant difference for age at onset between A and D1w (**p = 0.02) and, D1w and C (**p = 0.02); 2**B** Significant difference for age at onset between D1w and A2 (**p = 0.02) and A3 patients (**p = 0.02); **3A** Significant difference for disease duration between A and C (**p = 0.02), D1w and C (*p = 0.04), D1wo and C (**p = 0.02); **3B** Significant difference for disease duration between D1w and A1 patients (***p = 0.004).

All but two FSHD patients with a concomitant unrelated genetic disease (D1w) presented a D4Z4 repeat size ranging from 7 to 10 RU, with a mean repeat size significantly higher compared to category A patients (*p* < 0.0001), whereas age at onset, disease duration, and FSHD scores show no statistical significance difference. Subsequently, we compared patients from category A harboring 7 to 10 RU to patients from subcategory D1w and D1wo with the same repeat range. We found that D1w patients show a significantly earlier age at symptoms onset (*p* = 0.01) and a FSHD score significantly higher compared to category A patients (*p* = 0.005), while no differences were found regarding disease duration (*p* = 0.43) (Fig. 4). These results were of particular interest considering that D1wo patients showed no statistical difference when compared to category A in term of age at onset, FSHD score and disease duration (*p* = 0.33, *p* = 0.09, *p* = 0.12 respectively) (Fig. 4). We also made comparisons between D1w and D1wo with B and C categories. Both D1w and D1wo patients presented higher FSHD score compared to B and C (*p* < 0.0001 for D1w vs B and C, *p* = 0.001 for D1wo vs B and *p* = 0.0006 for D1wo vs C), and a longer disease duration compared to category C (*p* = 0.04 for D1w, *p* = 0.02 for D1wo). D1w patients showed also an earlier age at onset compared to category C (*p* = 0.02), while no differences were found when compared to category B (*p* = 0.1) and also when comparing D1wo with both B and C (*p* = 0.8, *p* = 0.35, respectively) (Fig. 4).

We then compared D1w patient carrying 7 to 10 RU to patients belonging to each subcategory A1, A2, A3 carrying the same repeat range. We have done also the same comparison for patients belonging to subcategory D1wo carrying 7 to 10 RU. D1w patients displayed similar age at onset (*p* = 0.9) and FSHD score (*p* = 0.13) but a shorter disease duration (*p* = 0.004) when compared to subcategory A1. They have a similar disease duration but an earlier age at onset and higher FSHD score when compared to A2 and A3 patients (*p* = 0.9 and *p* = 0.02 for both A2 and A3 for disease duration and age at onset and *p* = 0.003 vs A2 and *p* < 0.0001 vs A3 for FSHD score) (Fig. 4).

Regarding D1wo, we found no differences when comparing age at onset and disease duration with all subcategories A1, A2 and A3 (*p* ≥ 0.05 and *p* ≥ 0.1, respectively), whereas, when comparing FSHD score, D1wo patients showed mean values lower than A1 and higher than A2 and A3 (*p* = 0.006 and *p* = 0.04, respectively) (Fig. 4).

In summary, FSHD patients displaying uncommon features related to a concomitant unrelated genetic disease (D1w), represent in this cohort 8.9% of the total FSHD1 population (14/157). The large majority of these patients have 7 to 10 D4Z4 RU and display a more severe clinical phenotype than typical FSHD patients harboring the same D4Z4 repeat size range.

## Discussion

Facioscapulohumeral muscular dystrophy type 1 (FSHD1) exhibits significant variability both within and between families regarding age of onset, severity, and progression, complicating genotype-phenotype correlations. Generally, studies indicate that early onset is most often observed in FSHD1 patients with smaller D4Z4 repeats (1–3 RUs), while those with intermediate repeat sizes (4–6 RUs) tend to develop the classic form of the disease. Non-penetrant cases are more frequently associated with the largest repeat sizes (7–10 Rus) [7–11]. Nonetheless, exceptions exist; some are explained by variations in D4Z4 chromatin structure or the presence of somatic mosaicism for D4Z4 repeat contraction [12, 23]. Beyond variability in clinical expression and disease penetrance, a considerable number of FSHD patients present atypical phenotypes, including those without facial involvement, those with marked axial muscle weakness, and those with rare clinical or paraclinical features [9–11, 43–45].

In some patients with uncommon features, additional genetic investigations have revealed concomitant unrelated genetic diseases [31–38] (Supplementary table 1), suggesting the possibility that mutations in other genes may contribute to the clinical variability in FSHD. However, this is fundamentally different from the FSHD-intrinsic clinical variability caused by molecular heterogeneity from the FSHD1 locus itself, as exemplified by our previous findings of mutations in the *SMCHD1* gene acting as a genetic modifier of severity for FSHD1 patients [46] and of patients carrying a 18p deletion encompassing *SMCHD1* gene that in presence of a contracted 4qA D4Z4 allele display features of FSHD in association with uncommon features [47].

In the present study, most patients were classified in group A corresponding to patients exhibiting a typical FSHD clinical phenotype without uncommon features, with the largest number of patients in subgroup A2, showing upper and lower facial weakness and a FSHD score ≥1. These data are consistent with previous studies of the Italian patient population, in which the majority of patients were also classified as category A. On the other hand, in our study B and C categories were less represented while category D appeared to be more frequent [43–45].

The severity of facial weakness appears linked to overall clinical involvement, as previous studies have indicated. [43–45, 48] Patients in the A1 category, who exhibit more pronounced facial weakness, tend to have shorter D4Z4 repeat sizes, higher FSHD scores, longer disease durations, and an earlier onset of symptoms compared to those in A2 and A3 with milder facial involvement. This pattern suggests that facial weakness severity might correlate with disease duration, raising the possibility that classification within subcategories could change over time.

In our cohort, categories B1, B2, C1, and C2 were sparsely represented, as these less-affected or asymptomatic cases are typically identified in studies involving both affected and unaffected family members of an FSHD index case [43–45]. Since our study primarily focused on index cases and family members of D1 patients, the numbers in categories C1 and C2 were particularly low. Nonetheless, we observed that patients in categories B and C generally had a greater number of D4Z4 repeat units on their disease alleles compared to category A patients. Additionally, category C, representing asymptomatic or minimally affected patients, showed a female predominance, while category D, especially the D1w subgroup, showed a male predominance, differing from previous reports [43].

The number of patients initially classified in group D1 was relatively high (27 patients, 17% of total). The high prevalence observed may stem from a center bias, as our specialized FSHD facility likely attracts more complex or atypical cases. Consequently, our cohort may not represent the broader FSHD population, particularly those with typical presentations. This limitation should be considered, as it could affect the prevalence and distribution of clinical subtypes in our findings.

For 9 of the patients in group D1 (5.7%) we could find a clear relation with a non-genetic comorbidity causing the uncommon feature(s) (D1wo), for 14 of them a concomitant unrelated genetic disease (8.9%) was found and for 4 of them we could not find an explanation. We could not find in our cohort any patients belonging to group D2, maybe because this category appears to be very rare among patients with genetic diagnosis of FSHD [43, 45], especially index cases.

In subcategory D1w, we found 14/157 corresponding to 8.9% of patients with a concomitant and confirmed unrelated genetic disease. This frequency of patients with uncommon clinical features is consistent with what was found in previous studies using CCEF classification in terms of patients with uncommon features [45]. In these FSHD patients, we found a large and heterogeneous spectrum of rare genetic diseases including not only neuromuscular diseases like myotonic dystrophy type 1 and 2, mitochondrial myopathy associated to nuclear and mitochondrial DNA mutations and a case of calpainopathy (LGMD R1/2 A; MIM 253600), but also, unexpectedly, genetic diseases of the central nervous system (CNS) like Huntington’s disease and type 6 spinocerebellar ataxia.

It is intriguing that most of the concomitant unrelated genetic diseases associated with FSHD are repeat expansion diseases, including both neuromuscular and CNS disorders (11/14). It is tempting to speculate that the cooccurrence of FSHD1 with an unrelated repeat expansion disorder in these families is due to increased genetic susceptibility to repeat instability. Among the double troubles, we also identified a calpainopathy (LGMD R1/2 A; MIM 253600) and mitochondrial myopathies which are frequently considered in FSHD differential diagnosis because they have a very similar clinical phenotype.

Another very interesting finding is the observation that most FSHD1 patients with an unrelated genetic condition (but also more generally FSHD1 with uncommon features) have a disease allele of carried 7 to 10 D4Z4 RU. Repeat sizes of 8-10 D4Z4 RU are found with a frequency of 1–2% in the European population [49, 50] which may explain the relatively high frequency of double trouble cases having a combination of two rare genetic diseases. These findings are also in agreement with previous results showing that mutations in *SMCHD1*, acting as modifiers of disease severity, are preferentially found in FSHD1 patients with disease alleles in the high D4Z4 repeat range (9–10 RU) [12, 46]. Altogether these results suggest that FSHD associated to 7 to 10 D4Z4 repeats are more dependent on (epi)genetic or acquired modifiers to become clinically manifest. This would explain the higher frequency of non-penetrant carrier described in literature in this range of repeats [10, 45]. Smaller D4Z4 repeats (1–3 RUs) are generally linked to more severe, early-onset FSHD1, potentially obscuring atypical features like early gastrocnemius weakness due to extensive muscle involvement. This may impact the classification of patients into category D, as some atypical traits might be overlooked in individuals with pronounced FSHD. Additionally, patients with smaller D4Z4 (1–3 Rus) repeats may not have received the same level of comprehensive genetic testing as the D1w group, raising the possibility that secondary genetic diagnoses were missed in this subgroup. Although our findings indicate a higher prevalence of unrelated genetic disorders in patients with 7–10 repeats, the inconsistent testing limits our ability to conclude that those with smaller repeats are less likely to have a second genetic condition. Further studies with uniform genetic testing across all repeat sizes are needed to clarify this.

Based on our experience and the findings of this study, certain clinical features in FSHD patients may call for further genetic testing, especially when the presentation diverges from the typical FSHD phenotype. Additional testing should be considered in cases with unusual or early-onset symptoms, such as muscle weakness in non-typical areas (e.g., lower limbs or axial muscles) or marked asymmetry in muscle strength. Atypical neurological signs, like cognitive impairment, cerebellar signs, or movement disorders (e.g., chorea, ataxia), may also warrant further investigation, as they are not commonly associated with FSHD. Systemic complications, including respiratory insufficiency, cardiac abnormalities, or severe sensory deficits, suggest the need for extended genetic evaluation, particularly when these symptoms lack clear explanations from common comorbidities. A family history of neurological or neuromuscular disorders may indicate a separate genetic condition coexisting with FSHD1, supporting the need for comprehensive genetic assessment. When these atypical features are present, expanding the diagnostic workup with genetic testing, such as whole-exome sequencing or targeted panels for neuromuscular and neurological disorders, can provide valuable insights.

In conclusion, this study highlights the presence of unrelated genetic conditions in FSHD1 patients with atypical clinical features, particularly those with larger D4Z4 repeat sizes. These additional genetic conditions may intensify disease severity and contribute to phenotypic variability. In patients with larger repeat sizes, it is crucial to distinguish between variability directly due to the FSHD1 locus and clinical features resulting from unrelated genetic factors, to avoid confusing DUX4-related variability with unrelated influences. Recognizing the role of independent genetic factors in the intrafamilial and interfamilial variability of FSHD1 will be important not only for refining clinical management and genetic counseling but also to prevent clinical misclassification and improve patient stratification for therapeutic trials.

## Supplementary information

Supplementary Material

Dataset 1

## Data Availability

All data generated or analyzed during this study are included in this published article and its supplementary information files.
