# Supplementary material for: Double trouble: a comprehensive study into unrelated genetic comorbidities in adult patients with Facioscapulohumeral Muscular Dystrophy Type I
Source: Eur J Hum Genet. 2025 Jan 7;33(8):1006–14. doi: 10.1038/s41431-024-01770-0 (PMC12322215; doi:10.1038/s41431-024-01770-0)
Supplement: Supplementary file 1 — Supplementary Material [file 41431_2024_1770_MOESM1_ESM.docx]

**SUPPLEMENTARY MATERIAL**

**Family 1 (4A161, 10 RU)**

The proband (II,1), a 49 years old man, was referred to our hospital with a diagnosis of FSHD1 with a D4Z4 repeat array of 10 RU on a 4A161 allele inherited from the mother. Indeed, the 73 years old mother (I,2), was diagnosed with late onset FSHD since she developed later in life facial weakness, myalgia, camptocormia and had mildly increased CK levels (450 UI/L, nv <170) associated with the contracted FSHD1 allele. The proband described difficulty in raising arms starting at the age of 20 followed by the appearance of steppage gait and foot drop.

At neurological examination we noticed features typical of FSHD: *orbicularis oris* and *oculi* involvement, asymmetric weakness of shoulder girdle muscles with abduction limited to 90°, bilateral weakness of pectoralis major muscles, asymmetric weakness of tibialis anterior muscles and positive Beevor sign evocative of FSHD. We also noticed baldness and the presence of grip released myotonia suggesting a possible diagnosis of myotonic dystrophy type 1 (DM1), which was confirmed by the finding of 650 CTG expansion in *DMPK* (OMIM 605377). At follow-up after 6 months, we noticed bilateral cataract needing surgical intervention and cardiac conduction abnormalities requiring a pacemaker.

We also tested the 39 years old sister (II,2) who was asymptomatic but had an increase in CK value (250 UI/L, nv<170). She carried the 4A161 D4Z4 allele of 10 RU, but not the CTG expansion in *DMPK*. She was classified C2 in CCEF.

The father (I,1) was not tested because he died of sudden death at the age of 57, but he was likely to have DM1 as he was described as bald with troubles in releasing his hand after contraction and having had cataract surgery at the age of 35.

**Family 2 (4A161, 8 RU)**

The proband (I,2), was referred to our center at the age of 60 with a clinical diagnosis of FSHD based on the presence of scapular winging and facial involvement that she had developed since the age of 30. She had experienced a rapid decline starting at the age of 40, when she developed progressive lower limb weakness leading to the loss of ambulation and severe restrictive respiratory insufficiency. Family history reported a younger daughter (II,2) followed for severe cognitive impairment.

At clinical examination, we noticed bilateral asymmetric ocular ptosis in addition to *orbicularis oculi* and *oris* weakness, temporal muscles atrophy, diffuse distal and proximal weakness of upper and lower limbs.

Myotonia was not present, but the clinical phenotype was evocative of a complex disease with features of both FSHD and DM1. We examined the daughter (II,2) at the age of 34, who was diagnosed since early childhood of a severe cognitive impairment. She displayed facial weakness with features typical of DM1 (elongated face, ogival palate, ocular ptosis, temporal muscle atrophy), grip released myotonia but also weakness of orbicularis oculi, bilateral scapular winging and asymmetric tibialis anterior weakness.

The proband and the daughter were found to carry a CTG expansion of 650 and 950 CTG, respectively in *DMPK* gene, but also a 4A161 D4Z4 allele of 8 RU.

Following the diagnosis in both patients a severe dysrhythmic cardiomyopathy was discovered requiring pacemaker implantation. Unfortunately, the proband died the following year, due to a sudden aggravation of respiratory insufficiency. The daughter, who is now 39 years old, has lost ambulation.

**Family 3 (4A161, 8 RU)**

The proband (II,1), a 45 years old man, was referred to our center with a suspicion of FSHD since his mother, 67 years old (I,2) was diagnosed with FSHD (8 RU; 4A161) at the age of 64. She had developed orbicularis oculi and shoulder girdle muscle weakness since the age of 60 (CCEF category A, subcategory A3). The proband complained of myalgia, stiffness and shoulder girdle weakness since the age of 30. At clinical examination, we found facial weakness of *orbicularis oculis* and *oris*, asymmetric scapular winging, Beevor sign, asymmetric weakness of knee extension and tibialis anterior weakness consistent with a diagnosis of FSHD, but also distal arms weakness associated with grip released myotonia suggestive of DM1. The patient was found to carry the same D4Z4 4A161 allele as the mother and a 200 CTG expansion in the *DMPK* gene, confirming the clinical diagnosis of both FSHD1 and DM1. The mother did not carry the CTG expansion. The father died at the age of 60 of unknown causes. At the follow-up we discovered in the proband bilateral cataract requiring surveillance.

**Family 4 (4A161, 8 RU)**

The proband (I,2) was referred to our center at the age of 65 with a suspicion of myopathy due to the presence of steppage and weakness of shoulder girdle muscles from the age of 40 and she progressively developed severe axial weakness and distal lower leg weakness that led to loss of ambulation and restrictive respiratory insufficiency. She reported two sons having difficulty in raising their arms.

At clinical examination, she displayed asymmetric *orbicularis oculi* and *oris* weakness, proximal and distal weakness of upper and lower limb, and prominent axial weakness. She was found to carry a 4A161 allele of 8 RU consistent with the diagnosis of FSHD1. Following the diagnosis, both her sons were referred to our center. The older son (II,1), 37 years old, had unilateral scapular winging with no evidence of facial involvement and increased CK (460 UI/L, nv<170). He was classified in CCEF category B, subcategory B1. He carried the same 4A161 D4Z4 allele of 8 RU as his mother. The younger son (II,2), 32 years old, had bilateral scapular winging with abduction of the arm limited to 90°, but he also had cognitive impairment, *orbicularis oculi* and temporal muscle weakness, distal weakness in lower legs, grip released myotonia and tongue myotonia suggestive of DM1.

We found a CTG expansion in *DMPK* gene confirming the “double trouble” in both the mother (300 CTG) and the younger son (650 CTG) (II,2), but not in the older son (II,1). Since then, the proband died of the consequences of these diseases.

**Family 5 (4A161, 7 RU)**

The patient (II, 1) had a symptoms onset at the age of 60, with pain and progressive weakness of neck extension developed over one year approximately. Lower axial weakness then appeared, with difficulties in rolling from supine o prone. Weakness and myalgias were rather fluctuating, with periods of apparent improvement or worsening lasting few days to one week. On physical examination, he had head drop, neck flexor weakness (MRC=3), limited arm abduction (up to 90 degrees) and mild weakness of *orbicularis oris* muscle (he referred he was never able to whistle). Asymmetric hypotrophy of trapezius and mild scapular winging were also noticed, and he had not clinically evident myotonia.

His mother and maternal grandfather were reported to possibly have had camptocormia in late adulthood, family history was otherwise negative. Electromyography showed myopathic findings, with spontaneous activity, complex repetitive discharges and one myotonic discharge.

Based on clinical and instrumental findings, he was tested both for FSHD and DM2. The patient had a 4A161 D4Z4 allele of 7 RU and also one allele with an expanded CCTG repeat in the intron 1 of the *CNBP* locus, compatible with a diagnosis of DM2. None of the siblings was available for cascade testing. On last examination, at age 72, he was able to walk with a cane only for a few steps and used a walker or wheelchair for longer distances. He showed severe axial and diffuse proximal limb weakness, especially in the lower limbs.

**Family 6 (4A161, 7 RU)**

The proband (II, 2) was referred to our center at the age of 45 for diffuse myalgia, steppage gait and increased CK (556 UI/L, nv <200) developed since the age of 40. At clinical examination we noticed asymmetric *orbicularis oculi* weakness, mild shoulder girdle weakness with asymmetric scapular winging, positive Beevor sign and asymmetric *tibialis anterior* weakness, but also prominent calf hypertrophy. Electromyographic examination revealed a diffuse myopathic pattern and myotonic discharges at the proximal muscles. The cardiac and respiratory work out was unremarkable. He was found to carry a 4A161 allele of 7 RU. Because of the atypical phenotype we also checked for repeat expansions in *DMPK* and *CNBP* and we found an expansion of 500 CCTG in *CNBP* confirming the diagnosis of DM2.

The sister (II,1), 39 years old carried only the 7RU 4A161 allele and displayed asymmetric facial and shoulder girdle involvement typical of FSHD, which she developed since the age of 32. She was classified in CCEF category A, subcategory A3. The parents were not available for genetic testing because they were both deceased.

**Family 7 (4A161, 9 RU)**

The proband (I,1), a 66 years old man, experienced progressive weakness in axial muscles since the age of 50. At the age of 60 he was diagnosed with restrictive respiratory insufficiency and referred to our center.

Clinical examination showed asymmetric *orbicularis oculi* and *oris* weakness, severe axial weakness and symmetric distal weakness of quadriceps, hamstrings, and tibialis anterior muscles. The Beevor sign was positive. Lower limb osteotendinous reflexes were absent. He was found to carry a 4A161 allele of 9 RU consistent with the diagnosis of FSHD1. Because of the high CK level (1500 UI/L, nv <170) and the prominent respiratory involvement, he underwent a muscle biopsy which was evocative of a mitochondrial disease. An heterozygous *POLG1* variant NM_002693.3:c.3542G>A p.(Ser1181Asn) was found, which was already reported in literature as pathogenic (1).

The older son (II,1), 28 years old was also found to carry the same *POLG1* variant in association with sensory neuronopathy and with symmetric weakness of shoulder girdle muscles and pelvic muscles. He did not carry the contracted FSHD allele.

The younger son (II,1), 26 years old displayed only asymmetric scapular winging and weakness of shoulder anteflexion since the age of 20. His electromyography resulted normal, and he was found to carry the same 9 RU 4A161 allele as his father but not the *POLG1* variant.

**Family 8 (4A161, 9 RU)**

The proband (II,1), 35 years old man was referred to our center with a diagnosis of FSHD1 associated with a 4A161 allele of 9 RU.

In his family, the father, who died at 75 years of age, as well as several relatives on the father’s side, showed mild signs and symptoms of this disease and carried the same FSHD1 allele. The proband had severe muscle involvement starting in childhood and had lost his ambulation since the age of 20. At clinical examination, he displayed mild facial weakness, sensorineural hearing loss and severe proximal limb girdle weakness predominant in lower legs. *Tibialis anterior* muscles were also asymmetrically affected. He also complained of frequent episodes of migraine. Muscle biopsy was performed suggesting the presence of a mitochondrial disease. A heteroplasmic 3243A>G transition in the tRNALeu (UUR) (*MTTL1*) gene was found in both blood (40% of mutant mtDNA) and in muscle (60% of mutant mtDNA) derived DNA confirming the diagnosis of MELAS.

The mother (I,2), 63 years old, was also found to carry the same *MTTL1* variant in blood and muscle at lower levels of heteroplasmy (blood 20% of mutant mtDNA; Muscle 30% of mutant mtDNA). She complained of migraine and her CK levels were elevated (420 UI/L, nv<170) but her clinical examination was normal. The proband died of a severe stroke-like attack following a COVID19 infection.

**Family 9 (4A161, 10 RU)**

The proband (II,2), a 39 years old man, was referred to our center with a diagnosis of FSHD1, carrier of a 4A161 allele of 10 RU. His sister (II,1) and his mother (I,2) both experiencing myalgia and having a mild increase in CK values were known to have the same FSHD1 allele. They were both classified C2.

The patient started to experience symptoms at the age of 15 and, at clinical examination, displayed mild facial involvement including *orbicularis oculi* and *oris* muscle weakness, prominent asymmetric scapular winging associated with symmetric weakness of pelvic girdle muscles and hamstrings. He had CK values of 2500 UI/L. No cardiac or respiratory involvement was detected. Since he had a very high CK, we examined a muscle biopsy showing a dystrophic phenotype with normal immunostaining for all sarcolemmal membrane-associated proteins. Western blot analysis revealed a reduction of the 94 kD, the 60 kD and the 30kD bands of calpain 3. The patient was found to carry homozygous NM_000070.3:c.550delA p.(Thr184Argfs*36) variant in *CAPN3* (OMIM 114240).

**Family 10 (4A161, 6 RU)**

The proband (II,2), 27 years old man, was referred to our center for the presence of very high CK (2000UI/L, nv<170) and difficulty to raise his arms which he developed since the age of 18. The patient described also the presence of involuntary, irregular and unpredictable movements aggravated by stress, as well as apathy and depression. The mother of the patient (I,2), 56 years old, was also displaying involuntary movements, had difficulty in walking, talking and raising her arms.

At clinical examination, the proband showed mild facial involvement of *orbicularis oris muscles*, weakness of scapular muscles associated to a limitation of arm abduction at 90° and asymmetric weakness of *tibialis anterior* muscles, as well as a positive Beevor sign consistent with the diagnosis of FSHD. During examination, jerky involuntary movements of the feet and arms appeared. The patient was found carrying both a 4A161 allele of 6 RU and a 42 CAG trinucleotide expansion in the Huntington disease gene (*HTT*). The mother was found to carry the same 4A161 allele of 6 RU confirming the diagnosis of FSHD1 and a 38 CAG trinucleotide expansion in *HTT*. She died the following year of respiratory complications.

**Family 11 (4A161, 7 RU)**

The proband (II,1), a 26 years old man, was referred to our center for the finding of high CK (550UI/L nv 170), difficulty to raise his arms above shoulder and trouble walking and frequent falls. Her mother, 58 years old, was already followed in our department for FSHD associated to with a 4A161 allele of 7 RU. She displayed a classic phenotype including asymmetric facial and shoulder girdle weakness and positive Beevor sign, but no clinical involvement of lower legs. The father (I,1) died young in a car accident and was adopted.

At the clinical examination, the proband displayed weakness of *orbicularis oris* muscle and asymmetric scapular winging associated with a limitation in the abduction of the right arms above the shoulder to 90° and weakness of shoulder anteflexion prominent on the right arm; all suggestive of FSHD. No muscular weakness was found in lower legs explaining the falls. Instead, we found horizontal gaze-evoked nystagmus, intentional tremor of upper limbs and upper and lower limb ataxia suggesting a cerebellar syndrome. After excluding all possible autoimmune and paraneoplastic causes of cerebellar ataxia we focused on genetic assessments. The patient was found to carry the same FSHD1 allele of 7 RU as the mother, consistent with the diagnosis of FSHD1, but also a trinucleotide repeat expansion of 24 CAG units in *CACNA1A* associated with autosomal dominant spinocerebellar ataxia type 6 (SCA6). This variant was not present in the mother. Following the diagnosis the patient was treated with acetazolamide with a temporary reduction on the number of the falls. Unfortunately, more recently, dysarthria appeared, and the patient experience a worsening of ataxia and loss of ambulation.

**Supplementary table 1: FSHD “double trouble” reported in literature**

| References | FSHD | | | Other disease | | | **Comments** |
| --- | --- | --- | --- | --- | --- | --- | --- |
|  | Genetics | Age at onset | Distinctive features | Genetics | Age at onset | Distinctive features |  |
| Rudnik-Schoneborn et al (2008) doi:10.1016/j.nmd.2008.06.387 | 28kb;  4QA | 3YO | Cognitive impairment  Weakness predominant in facial and shoulder girdle muscles | *DYS*  c.4071+1 G>T) | 3YO | Hypotonia, proximal lower leg weakness, very high CK (7000 UI/L) | The father carried the 28kb allele and was asymptomatic. FSHD diagnosis was the first. Very high CK prompted the authors to search for another gene |
| Filosto et al (2008), doi:10.1016/j.nmd.2007.12.005 | 25kb;  4QA;  de novo | 34YO | Asymmetric shoulder girdle weakness, asymmetric weakness of *tibialis anterior ,* hyperlordosis, facial weakness | *mt-tRNA Leu (CUN)*  T12313C | NA | Muscle biopsy showed numerous ragged red fibers and cytochrome C oxydase negative fibers | This patient presented with no atypical features.  Fatigue and bilateral deafness are also seen in FSHD. FSHD diagnosis was the first. Muscle biopsy unusual features suggest the coexistence of 2 diseases |
| Ricci et al (2012), doi:10.1016/j.nmd.2011.12.001 | 35kb;  4QA161 | 45YO | Predominant scapular winging, hyperlordosis | *CAV3*  T78M | 53YO | Rippling phenomenon, muscle biopsy with low CAV3p immunostaining | The FSHD phenotype appeared earlier in life and oriented the diagnosis. Rippling phenomenon suggested the coexistence of another muscular disease |
| Masciullo et al (2013), http://dx.doi.org/10.1016/j.nmd.2013.02.002 | 24kb;  4QA161  de novo | Child  hood | Predominant asymmetric scapular winging, hyperlordosis | *DMPK*  500 CTG | 30YO | Dysmorphia, balding, ptosis, temporal  muscles wasting, handgrip and evoked myotonia. | Distal weakness is typical of both diseases, FSHD feature appeared earlier but the disease diagnosis was later. |
| Schreiber et al (2013), http://www.biomedcentral.com/1471-2350/14/92 | 19kb;  4QA | 50YO | Scapular winging, pectoralis muscle atrophy | *PMP22*  duplication | 20YO | Pes cavus, claw toes followed by weakness of dorsal interossei and thumb adduction, distal hypoesthesia, absence of osteo-tendinous reflexes | Distal lower leg weakness is a common feature of both diseases. FSHD symptoms leading to diagnosis appeared later in life. CMT diagnosis was earlier |
| Simeoni et al (2015), http://dx.doi.org/10.1016/j.jns.2014.12.009 | 35kb;  4Q not done | 45YO | Facial and upper girdle weakness | *CAPN3*  heterozygous mutation (c.266A N G, p.Y89C,  exon1) | 45YO | Weakness  of proximal lower limbs and the upper girdle  muscles (especially on the left side) with difficulties in performing  motor tasks | The clinical picture represents a mixture of the typical phenotypes of LGMD and FSHD. |
| Korngut et al (2008), doi:10.1016/j.nmd.2008.03.011 | 31kb;  4QA | Childhood | Symmetrical facial weakness, mild calf and  tongue hypertrophy.  Bilateral focal wasting of biceps and pectoralis major with an overlying axillary crease | *DMD*  hemizygous deletion  (c.367_368delGT, in exon 6) | 18 months | unable to walk, crawl, or pull to  stand and perform a shoulder shrug. Generalized hypotonia.  Deep tendon reflexes were absent. Plantar reflexes were downgoing.  Hig CK (2639 U/L) | Deltoids, biceps, triceps and all muscles of the lower extremities were  less than anti-gravity. No scapular winging |
| Scarlato et al (2014), DOI 10.1007/s00415-014-7606-2 | 25kb | 56YO | mild orbicular, scapuloperoneal and  axial muscle weakness with a limited arm abduction and  scapular winging, hyperlordosis, bilateral foot drop and proximal muscle weakness without spasticity | *SPAST*  (c.910_914delinsTAGG , p.Pro304*) | Late 20’s | Progressive spastic gait that became waddling but less spastic | The spastic gait was present in other family members and inherited as autosomic dominant trait |
| Rodolico et al (2020), https://doi.org/10.1016/j.ejpn.2020.05.006 | 4 Repeat Units  4Q not done | 11YO | Muscle weakness in orbicularis oculis and oris, waddling gait, lumbar hyperlordosis, weakness in neck flexors, pectoralis, triceps, wrist extensors, iliopsoas, quadriceps, and tibialis anterior muscles. Slightly increased serum Creatine Kinase (401 U/L) | 1.55 Mb deletion on chromosome 7q11.23. | 3YO | Thick lower lip vermilion, sloping shoulders, straight clavicles, bilateral scapular winging, scoliosis, moderate intellectual deficit (IQ 46) | The patient exhibited features from both WBS (early diagnosis) and FSHD (later diagnosis), with overlapping symptoms like scoliosis and lumbar hyperlordosis. |
| Rodolico et al (2020), https://doi.org/10.1016/j.ejpn.2020.05.006 | 6 Repeat Units  4Q not done | 12YO | Muscle weakness, scapular winging, asymmetric shoulders, wasting of pectoral and upper limb muscles, lumbar hyperlordosis, dorsal scoliosis, inability to raise arms above 45 degrees, increased CK levels (300-400 UI/L). | 1.55 Mb deletion on chromosome 7q11.23. | 16 months | Esotropia, periorbital puffiness, short nose, full cheeks, thick lower lip vermilion, small chin, bilateral temporal narrowing, moderate intellectual deficit (IQ 48) | The patient showed early signs of WBS with distinctive facial features and cardiovascular issues, while FSHD symptoms, such as muscle weakness and scapular winging, became apparent later. |
| Rodolico et al (2020), https://doi.org/10.1016/j.ejpn.2020.05.006 | 9 Repeat Units  4Q not done | 10YO | Muscle weakness, straight clavicles, axillary fold, severe lumbar hyperlordosis, prevalent upper limb wasting, difficulty raising arms, normal CK levels. | 1.55 Mb deletion on chromosome 7q11.23. | 11 months | Full cheeks, pouty lips, aortic and pulmonary valve stenosis, joint hypermobility, fair chest, moderate intellectual deficit (IQ 49). | The patient was diagnosed with WBS early due to facial dysmorphisms and cardiovascular problems. Over time, FSHD symptoms, including severe muscle weakness and lumbar hyperlordosis, developed, illustrating the progressive nature of FSHD in the context of WBS |

**REFERENCES**

1. Harrower T, Stewart JD, Hudson G, Houlden H, Warner G, O’Donovan DG, et al. POLG1 mutations manifesting as autosomal recessive axonal Charcot-Marie-Tooth disease. Arch Neurol. 2008;65(1):133–6.
